# The genome sequence of *Aegilops tauschii* subsp. *tauschii* Coss. accession Ent-118 (Poales: Poaceae)

**DOI:** 10.12688/wellcomeopenres.28080.1

**Published:** 2026-10-09

**Authors:** James Cockram, Richard Horsnell, Philip M. Howell, Bethany Love, Tally I. C. Wright, AEGIS Consortium, Wellcome Sanger Institute Tree of Life Management, Samples and Laboratory team, Wellcome Sanger Institute Scientific Operations: Sequencing Operations, Wellcome Sanger Institute Tree of Life Core Informatics team, Tree of Life Core Informatics collective

**Affiliations:** 1 Niab, Histon, Cambridge, England, CB24 9AT, UK

**Keywords:** Aegilops tauschii, Tausch’s goat grass, genome sequence, chromosomal, Poales

## Abstract

We present a genome assembly of *Aegilops tauschii* subsp. *tauschii* accession ‘Ent-118’ (diploid, 2 *n* = 2 *x* = 14, DD; Tausch’s goat grass; Streptophyta; Magnoliopsida; Poales; Poaceae). The assembly consists of two haplotypes with total lengths of 4,109.07 megabases and 4,009.71 megabases. Most of the haplotype 1 assembly (99.49%) was scaffolded into seven chromosomal pseudomolecules. Haplotype 2 was assembled to scaffold level. The mitochondrial genome assembly has a length of 415.5 kilobases and the plastid genome assembly has a length of 135.65 kilobases. Gene annotation of the haplotype 1 assembly by Ensembl identified 51,169 protein-coding genes. This assembly was generated as part of the Ancient Environmental Genomics Initiative for Sustainability (AEGIS) project.

## Species taxonomy

Eukaryota; Viridiplantae; Streptophyta; Streptophytina; Embryophyta; Tracheophyta; Euphyllophyta; Spermatophyta; Magnoliopsida; Mesangiospermae; Liliopsida; Petrosaviidae; commelinids; Poales; Poaceae; BOP clade; Pooideae; Triticodae; Triticeae; Triticinae; *Aegilops*; *Aegilops tauschii* Coss. (NCBI:txid37682)

## Background

*Aegilops tauschii* Coss. (2 *n* = 2 *x* = 14, DD genome) is a predominantly inbreeding diploid wild wheat species native to temperate and semi-arid regions of central, eastern and western Asia, from Turkey in the West to China in the East ( POWO, 2026). Via hybridisation with a cultivated tetraploid wheat in Neolithic farmers’ fields around 8,000 years ago, *Ae. tauschii* contributed the D subgenome of hexaploid bread wheat ( *Triticum aestivum* L., 2 *n* = 6 *x* = 42, AABBDD) ( Wang *et al.* , 2013), the world’s third most important cereal ( U.S. Department of Agriculture, 2026). Because *Ae. tauschii* harbours greater genetic diversity compared to that found across the D sub-genome of bread wheat, *Ae. tauschii* is regarded as a key component of the bread wheat secondary genepool ( Cox *et al.* , 2017). This diversity can be introgressed into bread wheat via several approaches, the most common of which is resynthesis: the creation of synthetic hexaploid wheat (SHW) by crossing *Ae. tauschii* with tetraploid durum wheat ( *T. turgidum* subsp. *durum* (Desf.) Husn.) followed by embryo rescue and chromosome doubling – enabling the growth of viable hexaploid plants for crossing into elite bread wheat genetic backgrounds (e.g. Gaurav *et al.* , 2022; Wright *et al.*, 2024).

Belonging to the Poaceae family, *Ae. tauschii* is a caespitose (tufted) annual grass species with erect or ascending stems of typically less than 40 cm in height, leaves of up to 15 cm long and 3 to 5 mm broad, and compact inflorescences consisting of around 7 to 10 spikelets. The two recognised sub-species can be distinguished by their inflorescence morphology, whereby subsp. *tauschii* has a cylindrical inflorescence composed of vertically elongated barrel-shaped spikelets, while subsp. *strangulata* (Eig) Tzvelev has a moniliform (necklace-like) appearance composed of wider, ventricose spikelets ( Eig, 1929). With intermediate forms between the two subspecies having been identified, molecular methods of classification have also been applied which group *Ae. tauschii* accessions into three lineages (L): L1 and L2 broadly correspond to subsp. *tauschii* and *strangulata,* respectively, while lineage L3 is rare and restricted geographically to Georgia ( Gaurav *et al.* , 2022). While the L2 lineage is the predominant donor of the bread wheat D sub-genome, minor contributions are evident from lineages L1 and L3 ( Gaurav *et al.* , 2022), potentially via admixture in the *Ae. tauschii* individual(s) involved in the speciation of bread wheat ( Wang *et al.* , 2024). A reference genome assembly for *Ae. tauschii* subsp. *strangulata* L2 lineage accession ‘AL8/78’ was released in 2017 ( Luo *et al.* , 2017), with the current update, Aet v6.0, released in 2025 ( Srikanta *et al.* , 2025). A recent *Ae. tauschii* pangenome has sequenced 46 accessions from across the species’ natural range ( Cavalet-Giorsa *et al.* , 2024a), comprising three chromosome-level assemblies ( *Ae. tauschii* accessions TOWWC0052 and TOWWC0116 from Turkmenistan, and TA2576 from Georgia) and 43 scaffold-level assemblies (data available at Dryad via Cavalet-Giorsa *et al.* , 2024b). An additional chromosome-level assembly has recently been made publicly available for the Iranian *Ae. tauschii* subsp. *strangulata* accession ‘Ent-076’ (GenBank assembly: GCA_965151655.1).

Here we describe a chromosome-level genome assembly for *Ae. tauschii* subsp. *tauschii* accession ‘Ent-118’ from the Yervan region of Armenia (Lat. 40.17°, Long. 44.49°). Previously assigned to *Ae. tauschii* lineage L1, this accession has been used as the D-genome donor for the creation of Niab synthetic hexaploid wheat accession ‘NIAB-SHW086’ ( Gaurav *et al.* , 2022). Seed for both ‘Ent-118’ and ‘NIAB-SHW086’ are available via the SeedStor, John Innes Centre, United Kingdom (https://www.seedstor.ac.uk), under GRU codes WS0477 and WS0029, respectively.

**Figure 1.**
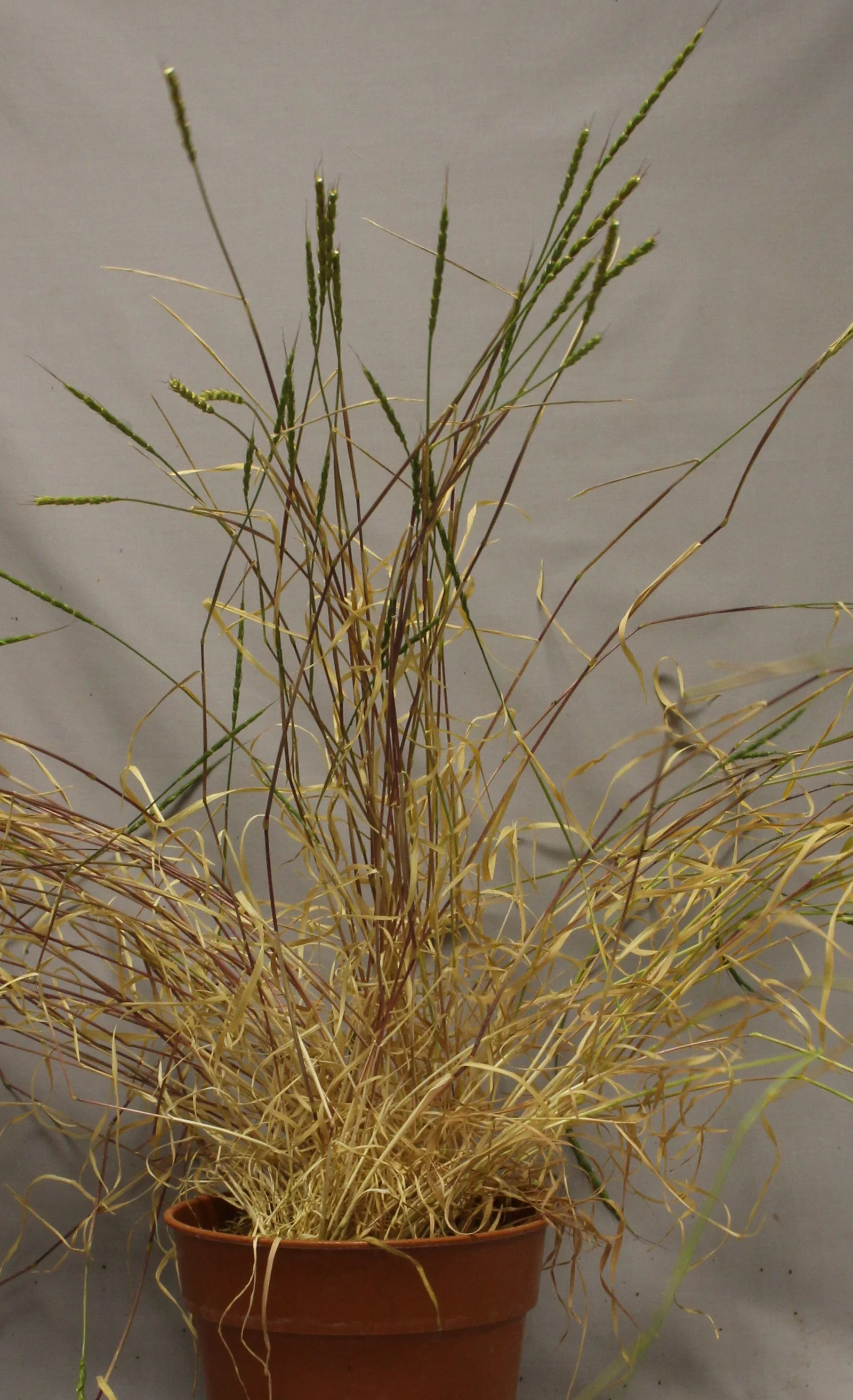
Glasshouse-grown plant of *Ae. tauschii* subsp. *tauschii* accession ‘Ent-118’.

## Methods

### Sample acquisition

A specimen of *Ae. tauschii* (specimen ID SAN20002203, ToLID lpAegTaus2; Figure 1) was used for genome, Hi-C and RNA sequencing. Seedling tissue was collected on 5 January 2024 from plants cultivated in the glasshouse at Niab, Cambridgeshire, UK. The specimen was collected by James Cockram.

### Nucleic acid extraction

High molecular weight (HMW) DNA extraction protocols developed by the Tree of Life Core Laboratory at the Wellcome Sanger Institute (WSI) are available on protocols.io ( Howard *et al.*, 2025). Tissue from the lpAegTaus2 sample was weighed and triaged to select the appropriate extraction workflow. HMW DNA was prepared for three PacBio HiFi libraries: 6771STDY15062983, 6771STDY15062984 and 6771STDY15062985.

The tissue input, HMW DNA extraction, downstream fragmentation and clean-up, and post-shearing quality-control measurements for each PacBio library are summarised in Table 1.

The concentration of the sheared and purified DNA was assessed with a NanoDrop spectrophotometer and a Qubit Fluorometer using the Qubit dsDNA High Sensitivity Assay kit. Fragment size distribution was evaluated by running the sample on the Femto Pulse system.

**Table 1.** DNA extraction and post-extraction processing for each PacBio library used for *Ae. tauschii*.

**PacBio library ID**	**Tissue preparation**	**HMW DNA extraction**	**Fragmentation and clean-up**	**Post-shearing QC**
6771STDY15062983	121.0 mg seedling; cryogenic bead beating	Automated Plant Organic Extraction	Fragmentation: Megaruptor®3 for LI PacBio; clean-up: Automated SPRI	Qubit 51.8 ng/μL; fragment size 14.0 kb
6771STDY15062984	105.0 mg seedlin; cryogenic bead beating	Automated Plant Organic Extraction	Fragmentation: Megaruptor®3 for LI PacBio; clean-up: Automated SPRI	Qubit 74.2 ng/μL; fragment size 23.1 kb

RNA was extracted from seedling tissue of lpAegTaus2 in the Tree of Life Laboratory at the WSI using the RNA Extraction: Automated MagMax™ *mir*Vana protocol. The RNA concentration was assessed with a NanoDrop spectrophotometer and a Qubit Fluorometer using the Qubit RNA Broad-Range Assay kit. RNA integrity was assessed using the Agilent RNA 6000 Pico Kit and Eukaryotic Total RNA assay.

### PacBio HiFi library preparation and sequencing

Library preparation and sequencing took place in the WSI Scientific Operations core. Libraries 6771STDY15062983, 6771STDY15062984 and 6771STDY15062985 were prepared with the SMRTbell Prep Kit 3.0 (Pacific Biosciences) according to the manufacturer’s instructions. The kit includes reagents for end repair/A-tailing, adapter ligation, post-ligation SMRTbell bead clean-up, and nuclease treatment. Size selection and clean-up were performed using diluted AMPure PB beads (Pacific Biosciences). DNA concentration was quantified with a Qubit Fluorometer v4.0 (Thermo Fisher Scientific) and the Qubit 1X dsDNA HS assay kit. Final library fragment size was assessed with the Agilent Femto Pulse Automated Pulsed Field CE Instrument (Agilent Technologies) using the gDNA 55 kb BAC analysis kit.

The libraries were sequenced in three runs on a Revio instrument (Pacific Biosciences, California, USA). Prepared libraries with a molarity greater than 2 nM were normalised to 2 nM, and 15 μL was used for making complexes. For libraries with a molarity below 2 nM, the available 10 μL library volume was used without normalisation. Primers were annealed and polymerases were bound to generate circularised complexes, following the manufacturer’s instructions. Complexes were purified using 1.2× SMRTbell beads, diluted to the Revio loading concentration of 200 to 300 pM, and spiked with a Revio sequencing internal control. Sequencing used Revio 25M SMRT cells. SMRT Link software (Pacific Biosciences), a web-based workflow manager, was used to configure and monitor the runs and to carry out primary and secondary data analysis.

### Hi-C

***Sample preparation and crosslinking***

Hi-C data were generated from the lpAegTaus2 seedling sample using the Arima-HiC v2 kit (Arima Genomics). Tissue was finely ground using the Covaris cryoPREP Dry Pulverizer (Covaris), and then subjected to nuclei isolation. Nuclei were isolated using a modified protocol based on the Qiagen QProteome Cell Compartment Kit (Qiagen), in which only the Lysis and CE2 buffers were used, with QIAshredder spin columns. After isolation, nuclei were fixed using formaldehyde to a final concentration of 2% to crosslink the DNA. The crosslinked DNA was then digested and biotinylated according to the manufacturer’s instructions. A clean-up step was performed with SPRIselect beads before library preparation. DNA concentration was quantified using the Qubit Fluorometer v4.0 (Thermo Fisher Scientific) and the Qubit HS Assay Kit, following the manufacturer’s instructions.

***Hi-C library preparation and sequencing***

Biotinylated DNA constructs were fragmented using a Covaris E220 sonicator and size-selected to 400 to 600 bp using SPRISelect beads. DNA was enriched with Arima-HiC v2 kit Enrichment beads. End repair, A-tailing, and adapter ligation were carried out with the NEBNext Ultra II DNA Library Prep Kit (New England Biolabs), following a modified protocol where library preparation occurs while DNA remains bound to the Enrichment beads. Libraries were amplified with KAPA HiFi HotStart mix and a custom Unique Dual Index (UDI) barcode set (Integrated DNA Technologies). Depending on sample concentration and biotinylation percentage determined at the crosslinking stage, libraries were amplified with 10 to 16 PCR cycles. Post-PCR clean-up was performed with SPRISelect beads. Libraries were quantified using the AccuClear Ultra High Sensitivity dsDNA Standards Assay Kit (Biotium) and a FLUOstar Omega plate reader (BMG Labtech).

Prior to sequencing, libraries were normalised to 10 ng/μL. Normalised libraries were quantified again to create equimolar and/or weighted 2.8 nM pools. Pool concentrations were checked using the Agilent 4200 TapeStation (Agilent) with High Sensitivity D500 reagents before sequencing. Libraries were sequenced on the Illumina NovaSeq X, generating paired-end 150 bp reads.

### RNA library preparation and sequencing

Libraries were prepared with the NEBNext® Ultra II Directional RNA Library Prep Kit for Illumina (New England Biolabs), following the manufacturer’s instructions. Poly(A) mRNA was isolated from 100 ng of total RNA using oligo(dT) beads, converted to cDNA, and uniquely indexed; 14 PCR cycles were performed. Libraries were prepared with an intended average fragment size of 100 to 300 bp. Libraries were quantified, normalised, and pooled equimolarly to a final concentration of 2.8 nM before dilution to 150 pM for loading. Sequencing was carried out on the Illumina NovaSeq X, generating paired-end reads.

### Genome assembly

Prior to assembly of the PacBio HiFi reads, a database of *k*-mer counts ( *k* = 31) was generated from the filtered reads using FastK. The *k*-mer frequency distribution was analysed with GenomeScope (version 2.1.0) ( Ranallo-Benavidez *et al.*, 2020), providing fitted point estimates of genome size, heterozygosity and repeat content.

The HiFi reads were assembled using Hifiasm in Hi-C phasing mode ( Cheng *et al.*, 2021, 2022), producing two haplotypes. Hi-C reads ( Rao *et al.* , 2014) were mapped to contigs using bwa-mem2 ( Vasimuddin *et al.*, 2019). Contigs were further scaffolded with Hi-C data in YaHS ( Zhou *et al.*, 2023), using the --break option for handling potential misassemblies. The scaffolded assemblies were evaluated using Gfastats ( Formenti *et al.* , 2022), BUSCO ( Manni *et al.*, 2021) and MerquryFK ( Rhie *et al.*, 2020). The organelle genomes were assembled using OATK ( Zhou *et al.*, 2025). The comparator for the plastid assembly was the chloroplast genome of *Aegilops tauschii* (NC_022133.1).

### Assembly curation

The assembly was decontaminated using the Assembly Screen for Cobionts and Contaminants ( ASCC) pipeline. The flat files and maps for curation were generated with TreeVal. Manual curation was conducted primarily in PretextView and HiGlass ( Kerpedjiev *et al.* , 2018). Scaffolds were visually inspected and corrected as described by Howe *et al*. (2021). The two haplotype assemblies were combined for curation. Manual corrections included 16 breaks and 33 joins. This reduced the scaffold count by 2.6% and reduced the scaffold N50 by 8.4%. The curation process is described at sanger-tol/curation-resources. A Hi-C contact map of the haplotype 1 assembly was generated with PretextSnapshot.

### Assembly quality assessment

The MerquryFK tool ( Rhie *et al.*, 2020) was run in a Singularity container ( Kurtzer *et al.*, 2017) to evaluate *k*-mer completeness and assembly quality for both haplotypes using the *k*-mer database ( *k* = 31) computed prior to genome assembly. The analysis outputs included assembly QV scores and completeness statistics.

The haplotype 1 assembly was assessed using BlobToolKit ( Challis *et al.*, 2020). PacBio reads were mapped to the assembly to derive sequence coverage, and BUSCO ( Manni *et al.*, 2021) was used to assess gene-set completeness. DIAMOND BLASTp searches of extracted BUSCO proteins and DIAMOND BLASTx searches of assembly sequences against UniProt Reference Proteomes ( Buchfink *et al.*, 2021), followed by BLASTn searches of sequences without DIAMOND BLASTx hits against the NCBI nt database ( Altschul *et al.*, 1990), supported taxonomic assignment. Coverage, sequence composition, taxonomic assignment and BUSCO results were integrated into a BlobDir for interactive inspection and visualisation. The snail and blob plots were rendered locally from this hosted BlobDir using BlobTk v0.8.3. The scaffold-length radial scale function for the snail plot was set to linear ( Challis & Blaxter, 2026). The blob plot used circular markers with area proportional to scaffold length ( Challis *et al.*, 2020).

## Genome sequence report

### Sequence data

The genome of a specimen of *Ae. tauschii* was sequenced using Pacific Biosciences single-molecule HiFi long reads, generating 191.92 Gb (gigabases) from 17.89 million reads. We assembled the genome from these reads. GenomeScope analysis using a *p* = 2 model gave a *k*-mer-based haploid genome size estimate of 4,211.13 Mb, with heterozygosity of 0.12% and repeat content of 69.33%; the full-model fit was 95.59% (Figure 2). This estimate guided expectations for the assembly. The sequencing data provided a reported 89× coverage of the genome. Hi-C sequencing produced 2,282.51 Gb from 7,557.99 million read pairs, which were used to scaffold the haplotype 1 assembly. RNA sequencing data were also generated and are available in public sequence repositories. Table 2 summarises the specimen and sequencing details.

**Figure 2.**
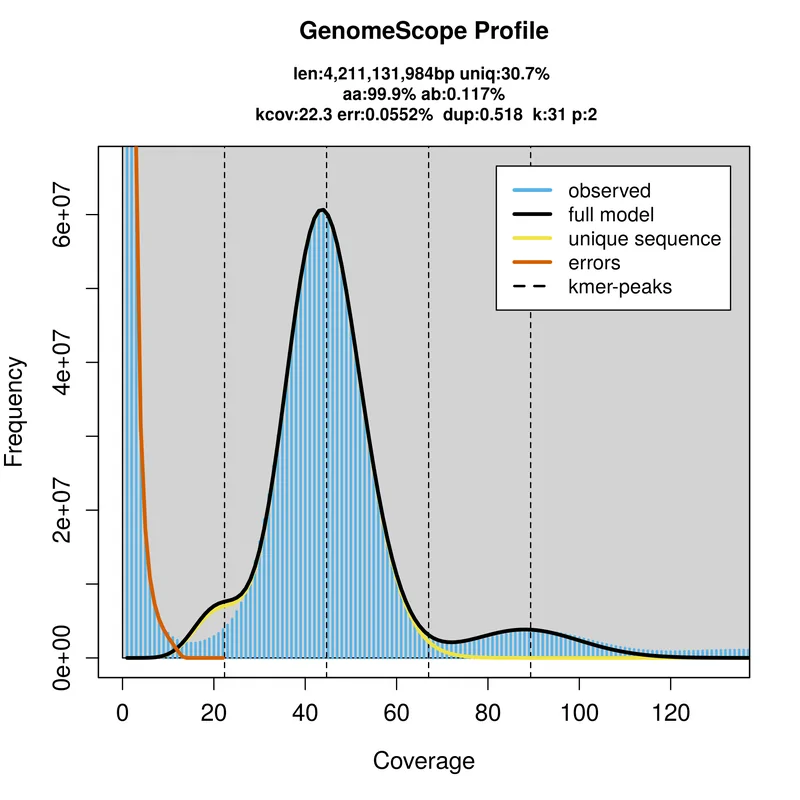
GenomeScope model fitted to the *k*-mer frequency distribution. The plot shows the observed and modelled *k*-mer spectra used to estimate genome size, heterozygosity and repeat content from unassembled sequencing reads.

**Table 2.** Specimen and sequencing data for *Ae. tauschii* (BioProject PRJEB81856)

**Platform**	**PacBio HiFi**	**Hi-C**	**RNA-seq**
**ToLID**	lpAegTaus2	lpAegTaus2	lpAegTaus2
**Specimen ID**	SAN20002203	SAN20002203	SAN20002203
**BioSample (source individual)**	SAMEA115133510	SAMEA115133510	SAMEA115133510
**BioSample (tissue)**	SAMEA115133667	SAMEA115133659; SAMEA115133667	SAMEA115133659; SAMEA115133667
**Tissue**	seedling	seedling	seedling
**Instrument**	Revio	Illumina NovaSeq X	Illumina NovaSeq X
**Library ID & run accession(s)**	6771STDY15062983: ERR13917215; 6771STDY15062984: ERR13917216; 6771STDY15062985: ERR13917217	ERR13925940; ERR13925942	ERR13962555; ERR13962557
**Read count total**	17.89 million reads	7,557.99 million read pairs	74.54 million read pairs
**Base count total**	191.92 Gb	2,282.51 Gb	22.51 Gb

### Assembly statistics

The genome was assembled into two haplotypes using Hi-C phasing. The haplotype 1 assembly was curated to chromosome level, while haplotype 2 was assembled to scaffold level. The haplotype 1 assembly has a total length of 4,109.07 Mb in 414 scaffolds (excluding organelle sequences), with 866 gaps, and a scaffold N50 of 583.02 Mb (Table 3). The haplotype 1 assembly has a scaffold auN of 588 Mb.

**Table 3.** Genome assembly statistics for *Ae. tauschii*

**Genome assembly**	**Haplotype 1**	**Haplotype 2**
**Assembly name**	lpAegTaus2.hap1.1	lpAegTaus2.hap2.1
**Assembly accession**	GCA_965151605.1	GCA_965151545.1
**Assembly level**	chromosome	scaffold
**Span (Mb)**	4,109.07	4,009.71
**Number of chromosomes**	7	-
**Number of contigs**	1,280	1,031
**Contig N50 (Mb)**	8.0	8.03
**Number of scaffolds (excluding organelle sequences)**	414	233
**Scaffold N50 (Mb)**	583.02	565.0
**Scaffold auN (Mb)**	588	575
**Longest scaffold length (Mb)**	656.32	649.43
**Organelles**	Mitochondrial genome: 415.50 kb; Plastid genome: 135.65 kb	-

Most of the haplotype 1 assembly sequence (99.49%) was assigned to 7 chromosome-level scaffolds. These chromosome-level scaffolds, confirmed by Hi-C data, are named according to size (Figure 3; Table 4).

The mitochondrial genome (length 415.5 kb, OZ235105.1) and plastid genome (length 135.65 kb, OZ235106.1) were also assembled. These sequences are included as contigs in the multifasta file of the haplotype 1 genome submission and as standalone records.

**Figure 3.**
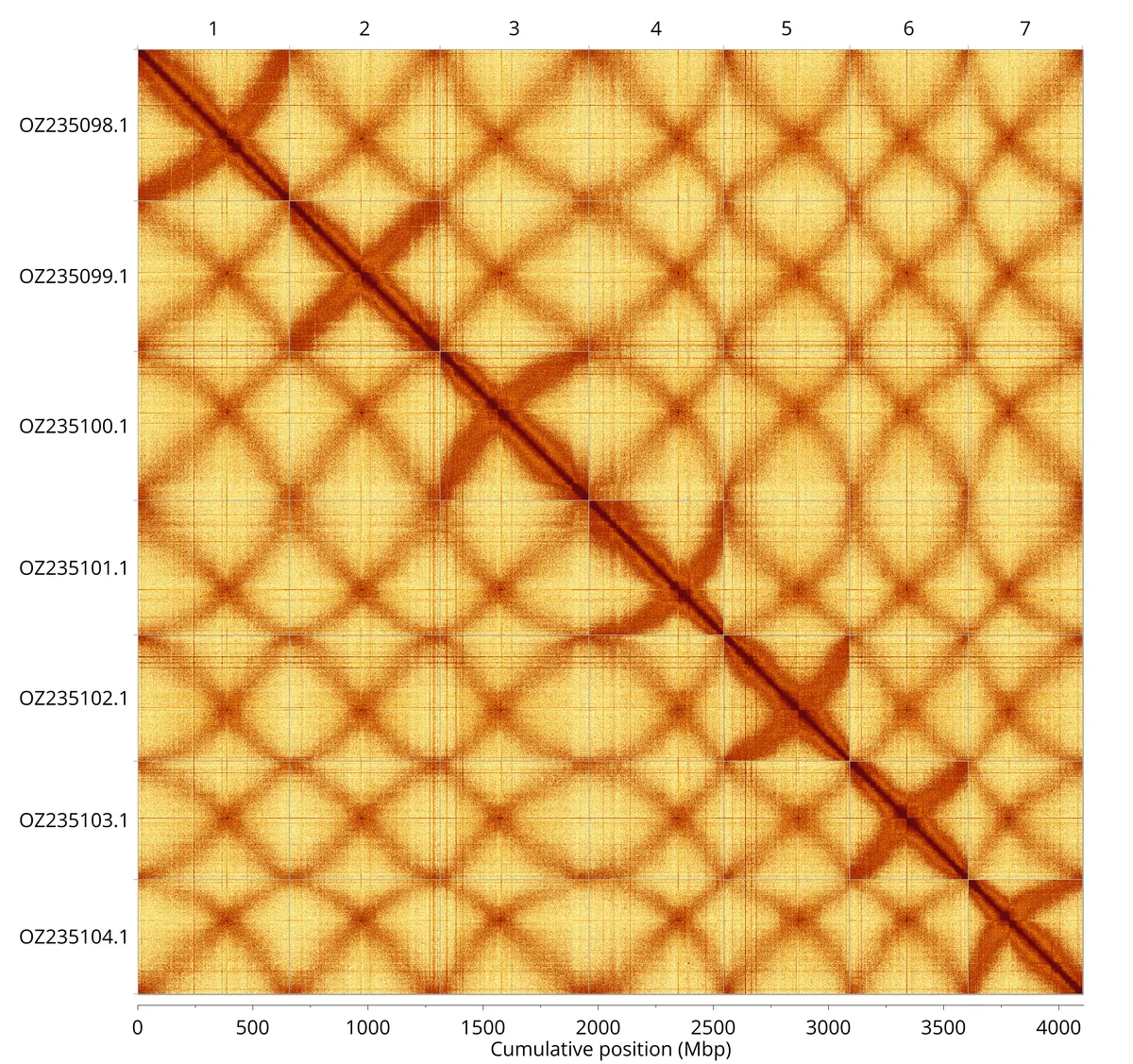
Hi-C contact map of the *Ae. tauschii* haplotype 1 assembly. Assembled chromosomes are shown in order of size and labelled along the axes, with a megabase scale shown below. The plot was generated using PretextSnapshot.

**Table 4.** Chromosomal pseudomolecules in the haplotype 1 genome assembly of *Ae. tauschii* lpAegTaus2

**INSDC accession**	**Molecule**	**Length (Mb)**	**GC%**
OZ235098.1	1	656.32	46.50
OZ235099.1	2	651.51	46
OZ235100.1	3	644.17	46
OZ235101.1	4	583.02	46
OZ235102.1	5	544.65	46.50
OZ235103.1	6	513.25	46
OZ235104.1	7	495.06	46

### Assembly quality metrics

For the haplotype 1 assembly, the estimated QV is 69.8, and for the haplotype 2 assembly, 69.9. When the two haplotypes are combined, the assembly achieves an estimated QV of 69.8. The *k*-mer completeness is 98.98% for the haplotype 1 assembly, 97.42% for the haplotype 2 assembly, and 99.15% for the combined haplotypes (Figure 4).

BUSCO analysis using the poales_odb10 reference set ( *n* = 4,896) identified 98.2% of the expected gene set (single = 94.5%, duplicated = 3.7%) in the haplotype 1 assembly. For the haplotype 2 assembly, BUSCO v.5.7.1 analysis identified 96.5% of the expected gene set (single = 92.8%, duplicated = 3.7%).

**Figure 4.**
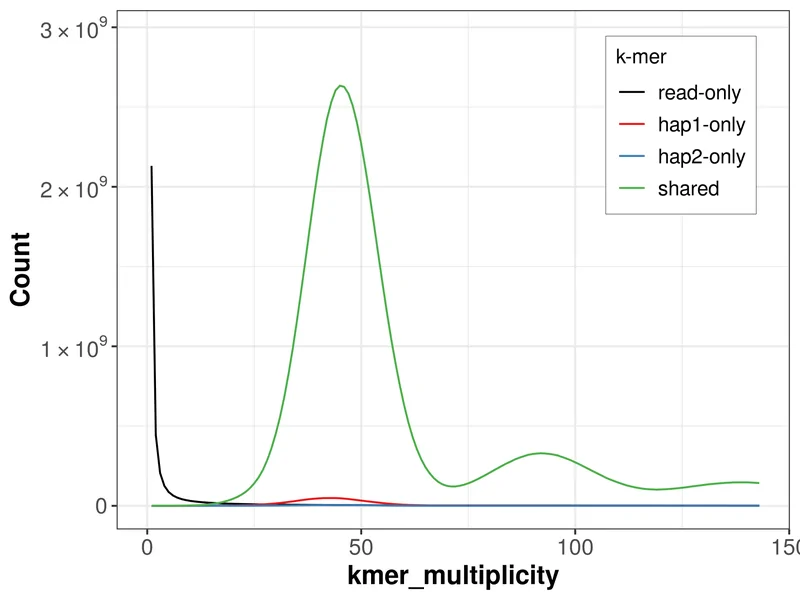
Evaluation of *k*-mer completeness using MerquryFK. This plot illustrates the recovery of *k*‐mers from the original read data in the haplotype 1 assembly, the haplotype 2 assembly and the combined haplotypes. The horizontal axis represents *k*‐mer multiplicity, and the vertical axis shows the number of *k*‐mers. The black curve represents *k*‐mers that appear in the reads but are not assembled, while the remaining curves correspond to *k*‐mers recovered in the assemblies shown.

The snail plot in Figure 5 provides a summary of assembly contiguity, cumulative scaffold count, sequence composition and BUSCO completeness for the haplotype 1 assembly ( Challis & Blaxter, 2026). The relative sizes of the longest scaffold, scaffold N50 and N90, together with the dark-grey scaffold-length distribution, show how much of the assembly is contained in long scaffolds. The outer composition track shows variation in GC, AT and N content, while the central track shows the cumulative scaffold count and the BUSCO inset summarises gene-set completeness.

The blob plot in Figure 6 displays scaffolds from the haplotype 1 assembly by GC proportion and base coverage. Marker area is proportional to scaffold length, and colour indicates taxonomic assignment ( Challis *et al.*, 2020).

**Figure 5.**
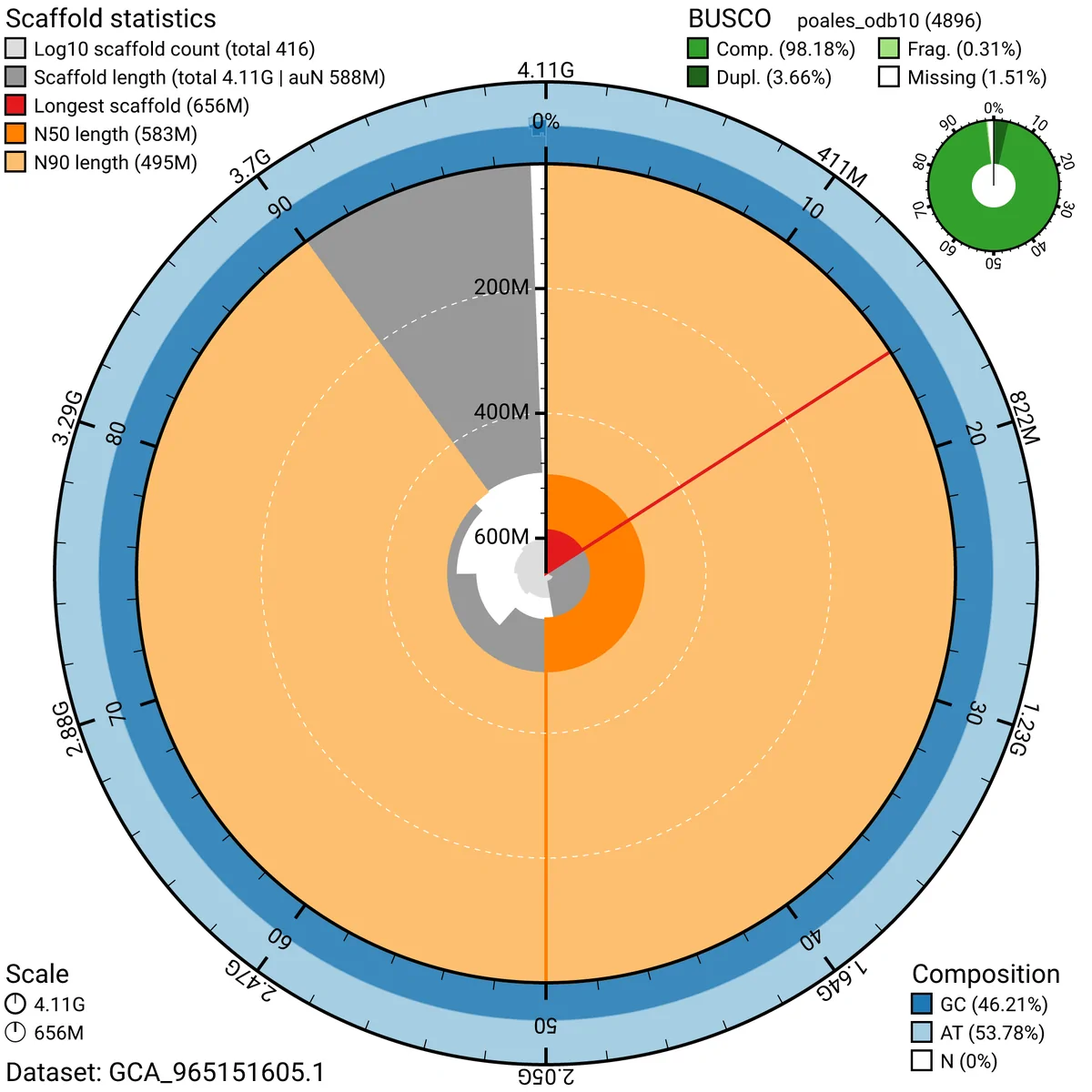
Assembly metrics for lpAegTaus2.hap1.1, the haplotype 1 assembly. The BlobToolKit snail plot provides an overview of assembly metrics and BUSCO gene completeness. The circumference represents the length of the haplotype 1 assembly, and the main plot is divided into 1,000 bins around the circumference. The outermost blue tracks display the distribution of GC, AT, and N percentages across the bins. Scaffolds are arranged clockwise from longest to shortest and are depicted in dark grey. Scaffold length is shown on a linear radial scale, scaled to the longest scaffold. Red shading indicates the longest scaffold, and the deeper orange and pale orange shading represent the N50 and N90 lengths. The summary statistics include scaffold auN, the area under the scaffold Nx curve. The light grey spiral at the centre shows the cumulative scaffold count on a logarithmic scale. A summary of complete, fragmented, duplicated, and missing BUSCO genes in the poales_odb10 set is presented at the top right. The assembly can be explored interactively in the BlobToolKit viewer.

**Figure 6.**
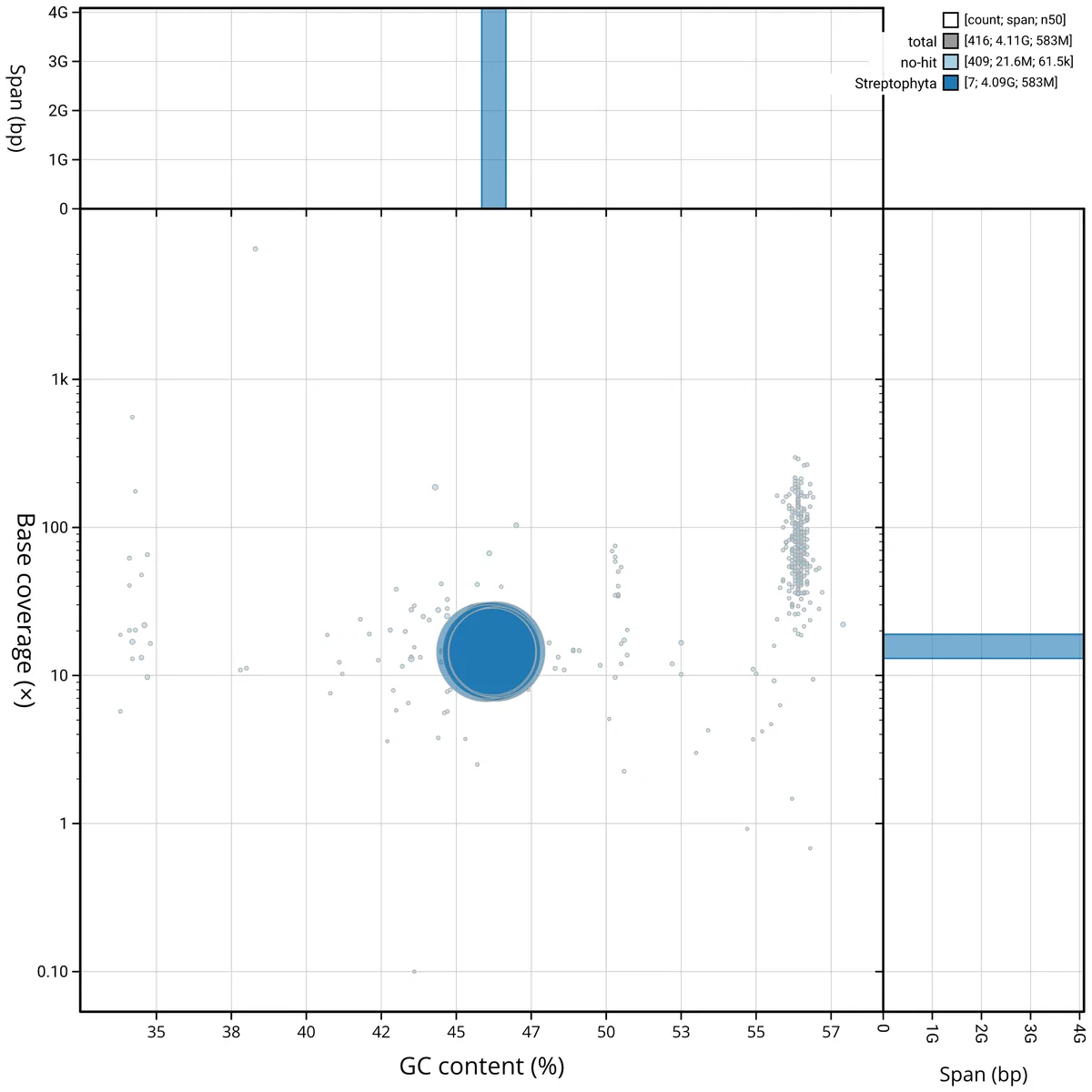
BlobToolKit blob plot for lpAegTaus2.hap1.1, the haplotype 1 assembly. The plot shows base coverage (vertical axis) and GC content (horizontal axis). Circles represent scaffolds; marker area is proportional to scaffold length, and colour indicates taxonomic assignment. The histograms along the axes display the total length of sequences distributed across different levels of coverage and GC content. The assembly can be explored interactively in the BlobToolKit viewer.

Table 5 presents assembly quality metrics alongside the Earth BioGenome Project (EBP) benchmarks described by Howe *et al.*. (2026). The three-part EBP metric calculated for the haplotype 1 assembly is **6.C.Q69**.

**Table 5.** Earth BioGenome Project summary metrics for the *Ae. tauschii* assembly

**Measure**	**Value**	***Benchmark***
EBP summary (haplotype 1)	6.C.Q69	*6.C.Q40*
Contig N50 length	8 Mb	*> 1 Mb*
Scaffold N50 length	583.02 Mb	*= chromosome N50*
Consensus quality (QV)	Haplotype 1: 69.8; haplotype 2: 69.9; combined: 69.8	*≥ 40*
*k*-mer completeness	Haplotype 1: 98.98%; Haplotype 2: 97.42%; combined: 99.15%	*> 90%*
BUSCO	Haplotype 1: C:98.2% [S:94.5%, D:3.7%], F:0.3%, M:1.5%, n:4,896; Haplotype 2: C:96.5% [S:92.8%, D:3.7%], F:0.4%, M:3.0%, n:4,896	*C > 90%*
Percentage of assembly assigned to chromosomes	99.49%	*≥ 90%*

**Notes:** The EBP summary uses log10(Contig N50); chromosome-level (C) or log10(Scaffold N50); Q (Merqury QV). BUSCO v5.7.1 using the poales_odb10 lineage dataset: C=complete; S=single-copy; D=duplicated; F=fragmented; M=missing; n=orthologues. For plants, the BUSCO benchmark uses total completeness (C = S + D) to accommodate biological duplication; this is a plant-specific adaptation of the EBP single-copy benchmark.

### Genome annotation report

The *Ae. tauschii* genome assembly (GCA_965151605.1) was annotated by Ensembl at the European Bioinformatics Institute (EBI). The annotation comprises 118,145 transcripts from 51,169 protein-coding genes and 44,130 non-coding genes. The average transcript length is 2,593.96 bp, with an average of 1.24 coding transcripts per gene and 3.32 exons per transcript. For further information about the annotation, please refer to the annotation page on Ensembl.

### Data availability

European Nucleotide Archive: *Aegilops tauschii* (goat grass). Accession number PRJEB81856; https://identifiers.org/ena.embl/PRJEB81856. The genome sequence is released openly for reuse. The *Ae. tauschii* genome sequencing initiative is part of the Sanger Institute Tree of Life Programme ( PRJEB43745) and AEGIS ( PRJEB80366). All raw sequence data and the haplotype assemblies have been deposited in INSDC databases. Raw data and assembly accession identifiers are reported in Table 2 and Table 3.

Pipelines used for genome assembly at the WSI Tree of Life are available at https://pipelines.tol.sanger.ac.uk/pipelines. Table 6 lists software versions used in this study.

**Table 6.** Software versions and sources

**Software**	**Version**	**Source**
BLAST	2.14.0	https://ftp.ncbi.nlm.nih.gov/blast/executables/blast+/
BlobToolKit	4.4.5	https://github.com/genomehubs/blobtoolkit
BlobTk (hosted BlobDir)	0.5.1	https://github.com/genomehubs/blobtk
BlobTk (snail and blob plot renderer)	0.8.3	https://github.com/genomehubs/blobtk
BUSCO	5.7.1; 5.5.0; 6.0.0; 6.1.0	https://gitlab.com/ezlab/busco
bwa-mem2	2.2.1	https://github.com/bwa-mem2/bwa-mem2
DIAMOND	2.1.8	https://github.com/bbuchfink/diamond
fasta_windows	0.2.4	https://github.com/tolkit/fasta_windows
FastK	1.2	https://github.com/thegenemyers/FASTK
GenomeScope	2.1.0	https://github.com/tbenavi1/genomescope2.0
Gfastats	1.3.6	https://github.com/vgl-hub/gfastats
Hifiasm	0.19.8-r603	https://github.com/chhylp123/hifiasm
HiGlass	1.13.4	https://github.com/higlass/higlass
MerquryFK	1.1.0-c1	https://github.com/thegenemyers/MERQURY.FK
Minimap2	2.28-r1209	https://github.com/lh3/minimap2
Oatk	0.9	https://github.com/c-zhou/oatk
MultiQC	1.14; 1.17 and 1.18	https://github.com/MultiQC/MultiQC
Nextflow	24.10.4	https://github.com/nextflow-io/nextflow
PretextSnapshot	0.0.6	https://github.com/sanger-tol/PretextSnapshot
PretextView	1.0.3	https://github.com/sanger-tol/PretextView
samtools	1.21	https://github.com/samtools/samtools
sanger-tol/ascc	0.1.0	https://github.com/sanger-tol/ascc
sanger-tol/blobtoolkit	v0.7.1	https://github.com/sanger-tol/blobtoolkit
sanger-tol/curationpretext	1.4.2	https://github.com/sanger-tol/curationpretext
Seqtk	1.4-r122	https://github.com/lh3/seqtk
Singularity	3.9.0	https://github.com/sylabs/singularity
TreeVal	1.4.0	https://github.com/sanger-tol/treeval
YaHS	1.2a.2	https://github.com/c-zhou/yahs

### Author information

Contributors represented by collective authorship are listed at the following links:

- Members of the Wellcome Sanger Institute Tree of Life Management, Samples and Laboratory team
- Members of Wellcome Sanger Institute Scientific Operations – Sequencing Operations
- Members of the Wellcome Sanger Institute Tree of Life Core Informatics team
- Members of the Tree of Life Core Informatics collective
- Members of the AEGIS Consortium

### Wellcome Sanger Institute – Legal and Governance

The materials that have contributed to this genome note have been supplied by a Tree of Life collaborator. The Wellcome Sanger Institute employs a process whereby due diligence is carried out proportionate to the nature of the materials themselves, and the circumstances under which they have been/are to be collected and provided for use. The purpose of this is to address and mitigate any potential legal and/or ethical implications of receipt and use of the materials as part of the research project, and to ensure that in doing so we align with best practice wherever possible.

The overarching areas of consideration are:

- Ethical review of provenance and sourcing of the material
- Legality of collection, transfer and use (national and international)

Each transfer of samples is undertaken according to a Research Collaboration Agreement or Material Transfer Agreement entered into by the Tree of Life collaborator, Genome Research Limited (operating as the Wellcome Sanger Institute) and in some circumstances other Tree of Life collaborators.
