# Supplementary material for: The genome sequence of Aegilops tauschii subsp. tauschii Coss. accession Ent-118 (Poales: Poaceae)
Source: Wellcome Open Res. 2026 Oct 9;11:836. [Version 1] doi: 10.12688/wellcomeopenres.28080.1 (PMC13687736; doi:10.12688/wellcomeopenres.28080.1)
Supplement: Peer review history

# Reviewer report: Reviewer response for version 1

**DOI:** 10.21956/wellcomeopenres.30896.r173499

**Published:** 2026-10-24

**Authors:** Volker P Brendel

**Affiliations:** 1 Indiana University Foundation, Bloomington, Indiana, USA

This is a perfectly executed genome project. The study is well motivated and makes a significant contribution to the scientific community.

 A minor comment relates to Table 6 and software provenance.My understanding is that the workflow was executed at the Wellcome Sanger Institute using their published Nextflow pipeline.If so, I am not sure what the table adds - would the reference to the pipeline version not take care of it? It could also be noted that the table often does not represent the latest versions of the programs (which of course will get more so in the coming years). Thus, reference to the frozen docker/apptainer container in the pipeline used would seem to be more efffective.

Are the datasets clearly presented in a usable and accessible format, and the assembly and annotation available in an appropriate subject-specific repository?

Yes

Are sufficient details of the sequencing and extraction, software used, and materials provided to allow replication by others?

Yes

Are the rationale for sequencing the genome and the species significance clearly described?

Yes

Are the protocols appropriate and is the work technically sound?

Yes

Reviewer Expertise:

computational genomics

I confirm that I have read this submission and believe that I have an appropriate level of expertise to confirm that it is of an acceptable scientific standard.

# Reviewer report: Reviewer response for version 1

**DOI:** 10.21956/wellcomeopenres.30896.r173502

**Published:** 2026-10-21

**Authors:** Bruno Contreras-Moreira

**Affiliations:** 1 Estación Experimental Aula Dei-CSIC, Zaragoza, Spain

The manuscript by J Cockram describes the assembly of the two genome haplotypes in tissue from Aegilops tauschii subsp. tauschii accession Ent-118. As haplotype 2 is scaffold level, only chr-level haplotype1 was annotated with the Ensembl pipeline, yielding 51K protein-coding genes, not far from the 49K reported in Ensembl for accession Ent-076, but significantly more that the 41K of T.urartu. I think the description of the annotated assembly is mostly clear and reproducible. Here are a few assorted comments:

 1 P5) Please explain why library 6771STDY15062985 is not in Table 1.

 2 P7) I know this statement is frequent in your genome reports, but to me it is hard to grasp: "the sequencing data provided a reported 89x coverage of the genome". Is this an estimate from kmer analysis or an observed average after mapping the original reads?

 3 P9) Please add a definition for auN, or cite a proper source. I can only find a hint on the legend of figure 5.

Are the datasets clearly presented in a usable and accessible format, and the assembly and annotation available in an appropriate subject-specific repository?

Yes

Are sufficient details of the sequencing and extraction, software used, and materials provided to allow replication by others?

Yes

Are the rationale for sequencing the genome and the species significance clearly described?

Yes

Are the protocols appropriate and is the work technically sound?

Yes

Reviewer Expertise:

Plant genomics, bioinformatics

I confirm that I have read this submission and believe that I have an appropriate level of expertise to confirm that it is of an acceptable scientific standard.

# Reviewer report: Reviewer response for version 1

**DOI:** 10.21956/wellcomeopenres.30896.r173496

**Published:** 2026-10-14

**Authors:** Nunzio D’Agostino

**Affiliations:** 1 University of Naples Federico II, Piazza Carlo di Borbone, Portici, Italy

This Genome Note presents a valuable genomic resource for Aegilops tauschii accession Ent-118, a lineage L1 accession used as a donor in synthetic hexaploidy wheat development. The authors report two haplotype assemblies, with haplotype 1 scaffolded into seven chromosomal pseudomolecules. The reported contiguity, consensus quality, k-mer completeness, and BUSCO completeness are strong. The inclusion of raw-read and assembly accession numbers, provenance information, and germplasm availability increases the resource’s usefulness.

**Major comments**

 The abstract states that the mitochondrial and plastid genomes were assembled. The manuscript does provide their lengths and accession numbers on page 9: 415.5 kb for the mitochondrial sequence (OZ235105.1) and 135.65 kb for the plastid sequence (OZ235106.1). It also states that they are included in the haplotype 1 multifasta submission and available as standalone records. Nevertheless, these results are described only briefly, and the reader cannot assess assembly completeness, structural reliability, or annotation status. Please include a dedicated organellar results paragraph or table reporting exact assembly size, sequence or component count, GC content, read coverage, unresolved gaps or ambiguities, and evidence supporting completeness.

 Explain the Oatk assembly and validation procedure, including relevant parameters and how organellar sequences were distinguished from nuclear copies.

 Please, explicitly state that the organellar sequences remain unannotated

 A brief comparison with the cited plastid reference NC_022133.1 and a relevant mitochondrial reference would help readers judge whether the reported sizes and structures are plausible.

 The Sequence data section on page 7 reports 191.92 Gb of HiFi sequence, a haploid genome estimate of 4,211.13 Mb, and 89× genome coverage. However, 191.92 Gb divided by 4.21113 Gb gives approximately 45.6× coverage relative to the estimated haploid genome. The reported 89× value therefore needs explanation or correction. Specify the denominator, the read set used, and whether the value denotes base coverage, k-mer coverage, or another quantity. Reconcile it with the GenomeScope profile in Figure 2 and the totals in Table 2.

 The text describes three PacBio libraries, but Table 1 lists only 6771STDY15062983 and 6771STDY15062984. Add the extraction, input tissue, fragmentation, cleanup, and QC details for 6771STDY15062985, or explain why they are unavailable.

 Clarify whether all DNA, Hi-C, and RNA datasets came from the same individual plant or from multiple plants of the accession. The manuscript refers to one specimen but also to seedling tissue collected from “plants,” and Table 2 lists two tissue BioSamples for Hi-C and RNA-seq. Explain how the tissue records relate to the source individual and libraries.

 The laboratory procedures are described in useful detail, and Table 6 lists software versions. However, important computational decisions remain insufficiently specified. Report the HiFi filtering criteria, the Hifiasm settings used for Hi-C phasing, the Hi-C alignment and filtering procedure, and the configuration used for scaffolding, decontamination, and organelle assembly.

 Explain the statement that the haplotype assemblies were combined for curation. Were both haplotypes scaffolded and inspected together, and how were the corrected sequences subsequently assigned to each output? State whether the 16 breaks and 33 joins refer to haplotype 1, haplotype 2, or their combined set, and provide the corresponding before-and-after statistics.

 Table 6 lists four BUSCO versions and several MultiQC versions; identify which versions generated each reported analysis so the published metrics can be reproduced.

 The two assemblies differ in length by approximately 99.36 Mb, and their reported BUSCO completeness is 98.2% and 96.5%. Both are shorter than the GenomeScope haploid estimate. Briefly discuss whether these differences reflect unresolved sequence, filtering or curation decisions, uncertainty in the genome-size estimate, or plausible biological variation. Do not assign a biological cause without supporting evidence.

 Page 9 states that pseudomolecules are named by size, and Table 4 labels them 1–7. Please make clear that these are size-ranked scaffold identifiers and are not established chromosome homology assignments. For a species with existing reference assemblies and a direct relationship to the wheat D genome, a correspondence table to a cited Aegilops reference would improve usability. Provide the assignment method if such correspondence is established and indicate pseudomolecule orientation. If it has not been determined, state this explicitly to prevent users from assuming that molecule 1 corresponds to chromosome 1D.

 The Methods describe RNA extraction and RNA-seq library preparation and sequencing and identify seedling tissue from glasshouse-grown material. However, the sampled organs or tissue composition, seedling age or developmental stage, growth conditions, collection timing, and any treatments are not adequately specified. Please provide these details, the number of plants contributing to each sample, any pooling, and the relationship between RNA libraries and the two tissue BioSamples. State whether the libraries represent separate biological samples or technical sequencing of the same material.

 The purpose and subsequent use of the transcriptomic data are also unclear. Presumably, these data supported genome annotation, but this should be stated explicitly rather than inferred. Describe whether the reads were used for transcript alignment, transcript assembly, gene prediction, splice-junction validation, or another purpose, and report relevant preprocessing, mapping, evidence integration, and quality-control procedures. If these RNA-seq datasets were not used in annotation, state that and explain their intended role as a reusable resource.

 The annotation summary on page 14 does report 51,169 protein-coding genes, 44,130 non-coding genes, 118,145 transcripts, and several average transcript or gene-structure statistics. The major omission is the annotation methodology and validation, rather than the complete absence of annotation results. Identify the Ensembl release and annotation pipeline, software versions and parameters, repeat identification and masking strategy, integration of transcript and protein evidence, and criteria for filtering or selecting gene models. Explain functional annotation procedures and databases.

 Include stable links to the GFF3/GTF, transcript, and protein files, and state whether only haplotype 1 has been annotated.

 As this article is presented as a Genome Note, please add a brief section near the end of the manuscript outlining how the scientific community can reuse and benefit from the genomic resources. Explain the accession’s contribution to existing Aegilops tauschii resources and identify realistic applications, such as comparison of L1 and L2 diversity, analysis of the wheat D-genome donor, candidate-gene discovery, marker development, and evaluation of introgressions in synthetic hexaploid wheat. Distinguish potential applications from findings demonstrated in this study.

**Minor comments**

 1. Correct “seedlin” in Table 1 and “Yervan” in the Background. Standardize accession naming and the capitalization of Oatk throughout.

 2. Give more informative captions for Figures 2 and 4. Define the reported GenomeScope parameters and explain the haplotype-specific and shared k-mer curves in terms accessible to users of the resource.

 3. State whether organellar contigs are included in each quality analysis. The 414 nuclear scaffolds in Table 3 and 416 sequences in the snail plot appear consistent with inclusion of the two organellar sequences in the latter; make that distinction explicit.

 4. Add a brief explanation of the unplaced sequence fraction and the contamination-screening outcome, including any sequences removed or retained as uncertain. The blob plot alone does not fully document those decisions.

Are the datasets clearly presented in a usable and accessible format, and the assembly and annotation available in an appropriate subject-specific repository?

Partly

Are sufficient details of the sequencing and extraction, software used, and materials provided to allow replication by others?

Partly

Are the rationale for sequencing the genome and the species significance clearly described?

Yes

Are the protocols appropriate and is the work technically sound?

Yes

Reviewer Expertise:

Bioinformatics and genomics

I confirm that I have read this submission and believe that I have an appropriate level of expertise to confirm that it is of an acceptable scientific standard, however I have significant reservations, as outlined above.

# Reviewer report: Reviewer response for version 1

**Published:** 2026-10-09

**Authors:** Automated Benchmarking

**Affiliations:** 1 Wellcome Sanger Institute, Hinxton, UK

As this article is a Genome Note, Automated Benchmarking has been used to determine whether the reported assembly passes four defined checks established by the Earth BioGenome Project: the raw and assembled data are publicly available, the Earth BioGenome Project three-part metric is met, the k-mer completeness exceeds 90%, and the BUSCO score passes the stated threshold. For Aegilops tauschii (lpAegTaus2), all four checks passed. Data available: pass. ENA study PRJEB81856, raw-read accessions ERR13917215, ERR13917216, ERR13917217, ERR13925940, ERR13925942, ERR13962555, and ERR13962557, and assembly accessions GCA_965151605.1 and GCA_965151545.1 are public. Earth BioGenome Project three-part metric: pass. Haplotype 1 is reported as 6.C.Q69, meeting the 6.C.Q40 reference standard. k-mer completeness: pass. The combined haplotype assemblies have 99.15% k-mer completeness, exceeding the Earth BioGenome Project target of 90%. BUSCO: pass. Haplotype 1, which is proposed as the reference assembly for this species, has 98.2% complete BUSCOs (94.5% single-copy and 3.7% duplicated). BUSCO software version: 5.7.1; lineage dataset: poales_odb10. It meets the plant-specific target of greater than 90% complete BUSCOs. This decision uses values reported in the Genome Note and public accession records.

Reviewer Expertise:

NA

I confirm that I have read this submission and believe that I have an appropriate level of expertise to confirm that it is of an acceptable scientific standard.
